# Characteristics and homogeneity of N6-methylation in human genomes

**DOI:** 10.1038/s41598-019-41601-7

**Published:** 2019-03-26

**Authors:** Clare E. Pacini, Charles R. Bradshaw, Nigel J. Garrett, Magdalena J. Koziol

**Affiliations:** 1 Wellcome Trust Cancer Research UK Gurdon Institute, University of Cambridge, Cambridge, CB2 1QN UK; 2 Department of Zoology, University of Cambridge, Cambridge, CB3 3EJ UK

## Abstract

A novel DNA modification, N-6 methylated deoxyadenosine (m6dA), has recently been discovered in eukaryotic genomes. Despite its low abundance in eukaryotes, m6dA is implicated in human diseases such as cancer. It is therefore important to precisely identify and characterize m6dA in the human genome. Here, we identify m6dA sites at nucleotide level, in different human cells, genome wide. We compare m6dA features between distinct human cells and identify m6dA characteristics in human genomes. Our data demonstrates for the first time that despite low m6dA abundance, the m6dA mark does often occur consistently at the same genomic location within a given human cell type, demonstrating m6dA homogeneity. We further show, for the first time, higher levels of m6dA homogeneity within one chromosome. Most m6dA are found on a single chromosome from a diploid sample, suggesting inheritance. Our transcriptome analysis not only indicates that human genes with m6dA are associated with higher RNA transcript levels but identifies allele-specific gene transcripts showing haplotype-specific m6dA methylation, which are implicated in different biological functions. Our analyses demonstrate the precision and consistency by which the m6dA mark occurs within the human genome, suggesting that m6dA marks are precisely inherited in humans.

## Introduction

DNA modifications directly alter DNA, thus any such alterations are likely to have major effects on the genome, transcription and human health. DNA modifications affecting the DNA base deoxycytidine have been studied extensively in humans, revealing fundamental roles in regulating gene transcription and through that affecting many human diseases, particularly cancer^1–3^. 5-methyl-deoxycytidine (m5dC) is the most characterized DNA modification, in which a methyl group is directly attached at the carbon-5 position of the DNA base deoxyadenosine. In prokaryotes, other DNA modifications have also been studied widely, such as N-6-methyl-deoxyadenosine (m6dA)^4–9^. m6dA is a DNA modification in which a methyl group is linked to the adenosine base at the nitrogen-6 position of the DNA base deoxyadenosine. In prokaryotes, m6dA is known to have important functions^4–9.^

Recently, m6dA has been identified and described in eukaryotes such as flies^10^, algae^11^, worms^12^, frogs^13^, zebrafish^13^, mouse^13,14^, humans^13,15^, rat and plants^15^. Following these discoveries, m6dA has also been confirmed to be present in mouse^16,17^, pig, zebrafish^18^, yeast^19^, ciliate^20^ and humans^21,22^. Studies have now focused on identifying m6dA modifying enzymes to study m6dA function. Recent studies suggested that the mammalian enzyme AlkBH1 demethylates m6dA^16,23,24^. Removal of AlkBH1 in mouse embryonic stem cells decreased transcription of some genes near m6dA sites^16^. However, another study could not reproduce the demethylating AlkBH1 function^25^, demonstrating that further work is required to fully elucidate the role of AlkBH1 in regulating m6dA. Recently, the mammalian enzyme N6AMT1 was shown to be a m6dA methylase^23^. N6AMT1 knockout mice had lower m6dA levels and promoted tumorigenesis, while m6dA abundance was reduced in gastric and liver cancers^23^. However, in the brain cancer glioblastoma, a higher m6dA level was observed^24^, suggesting that any misregulation of m6dA levels may result in disease. Also, m6dA was found at decreased levels in type two diabetes mellitus^15,21,24^.

Studying m6dA is critical for a better understanding of human diseases. As a first important step, a precise map of m6dA sites within the human genome is needed. This can be established using Single Molecule Real-Time sequencing (SMRT-seq), which allows the detection of m6dA at nucleotide level resolution^26^. This technology sequences DNA by detecting fluorescent nucleotides that specifically base pair to each complementary nucleotide of the DNA library. The sequence of fluorescent nucleotides provides the DNA sequence whilst the duration of the incorporations, referred to as the inter-pulse duration (IPD)^27,28^, is also measured. A nucleotide modification can significantly change the time it takes for a fluorescent nucleotide to be incorporated. Changes in the IPD value from the expected level, where DNA is not modified, are represented by the IPD ratio. Different modifications and sequence contexts can produce different IPD values and kinetic signatures^27,28^. However, changes in IPD ratios within a sequence context can be indicative of a particular modification. As errors occur during sequencing, an increased coverage can ensure that false positives and negatives are reduced. To increase coverage and decrease errors, SMRT-seq is performed on circular DNA libraries. This circular structure allows the same fragment to be sequenced multiple times, significantly improving coverage of the sequenced DNA fragment, and as such its sequence and m6dA detection^16,29^. In fact, it was determined that for reliable m6dA detection in mammals, a minimum coverage of 20x is required^16^. Due to this low coverage requirement in detecting m6dA at nucleotide resolution, SMRT-seq is particularly well suited to establish a precise map of m6dA within genomes.

m6dA was identified at nucleotide resolution genome-wide in prokaryotes^30,31^, worms^12^, ciliate^20^, fungi^19^ and algae^22,32^. Although m6dA locations were also identified with SMRT-seq in mouse, relaxed IPD cutoffs were used and the study was limited by only considering regions associated with the chromatin mark H2A.X^16^. More recently, m6dA was also identified by SMRT-seq in the human genome^22,23^. Analysis revealed interesting human m6dA characteristics, such as m6dA enrichment within exons that show increased transcription^22,23^. This suggests that m6dA could directly regulate transcription. Transcription is already well known to be regulated by the DNA modification deoxycytidine 5-methylation (m5dC). m5dC can be heterogenous between chromosome pairs within the same cell-type or cell line^33–37^. This epigenetic ‘heterogeneity’ at a specific genomic location is believed to cause differential gene expression, which is for example required for establishing and maintaining a cell’s identity and generating cell subpopulations within the same cell type or cell lines^38–40^. Not surprisingly, heterogeneity is particular important in diseases such as human cancers, making cancer cells difficult to eliminate. To improve treatments, a better understanding of epigenetic heterogeneity will be important^41^.

Despite new m6dA insights, m6dA heterogeneity between different cell types, between cells within the same cell population, and between chromosome pairs has so far not been investigated. However, a better understanding of m6dA heterogeneity is essential: First, m6dA is a low abundance DNA modification. If these low abundance m6dA marks are placed at some precise nucleotide positions consistently, this would suggest conservation and important m6dA regulatory functions in these genomic locations. Second, knowing if or where m6dA is homogeneous would facilitate identifying and studying m6dA marks at these homogenous and precise locations, as associated phenotypes are most likely be most penetrant. Thirdly, identifying precise sites at which m6dA occurs, and distinguishing between heterogenous and homogenous m6dA marks will open up opportunities to target for therapeutic purposes only specific m6dA locations that are associated with diseases.

Here, we identify m6dA within different human genomes at nucleotide resolution, reveal novel m6dA features and specifically investigate m6dA heterogeneity between different cell types, between cells within the same cell population, and between chromosome pairs.

## Results

### Features of m6dA in different human genomes

To identify m6dA sites in the human genome, we made use of published human genome SMRT-seq datasets. We selected two published human SMRT-seq datasets with a high genome-wide coverage, AK1, an immortalized lymphoblastoid cell line from a Korean male^42,43^, and CHM1, a hydatidiform mole female cell line^44^. Although CHM1 has a coverage of 54x^44^, and AK1 a coverage of 101x^42,43^, they are comparable, as AK1 is diploid and CHM1 is haploid^42,43^. To reliably identify m6dA sites, we only focused on regions with high sequence coverage from the same DNA library fragment (>20) when it showed significant IPD ratios (≥4)^22^.

**Figure 1:**
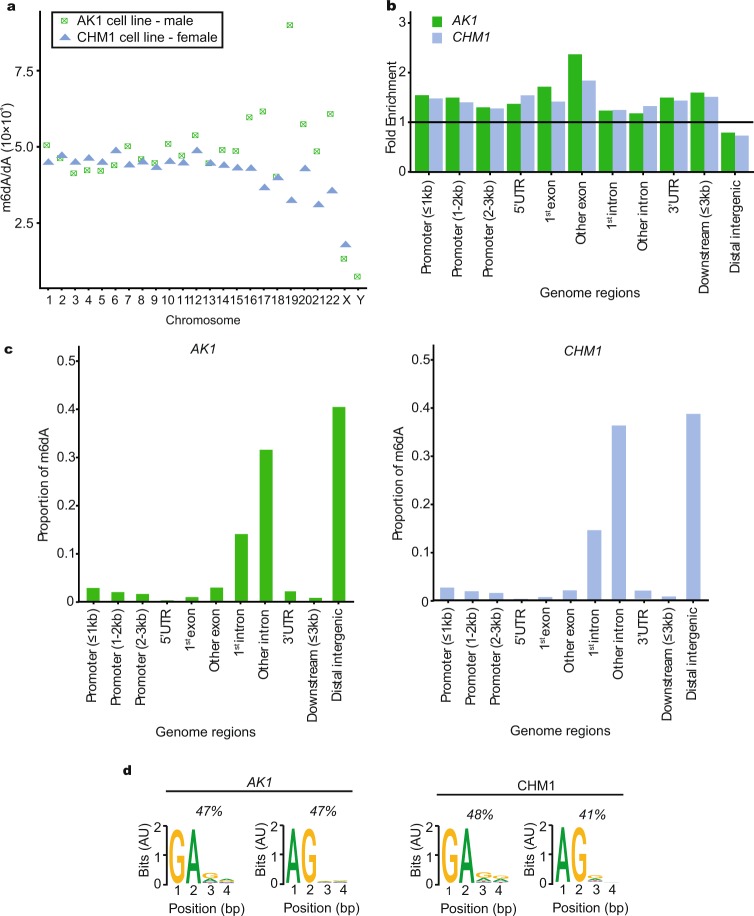
Genomic location of m6dA. (**a**) Proportion of m6dA versus unmethylated deoxyadenosines (m6dA/dA) per chromosome is shown. () CHM1, () AK1 cell lines. (**b**) Fold m6dA/dA enrichment in different genomic regions. The horizontal line indicates the expected levels of m6dA/dA for each region. () CHM1, () AK1 cell lines, (UTR) untranslated regions. (**c**) Proportion of m6dA in distinct genomic regions for AK1 and CHM1 cell lines. (UTR) untranslated regions. (**d**) Human m6dA motifs identified show strong abundance for GA or AG sequences in AK1 and CHM1 cell lines (P-value < 2.2 × 10^−16^, Fishers exact test). (AU) arbitrary units.

Using these filters, we identified 74,345 m6dA sites in CHM1 and 80,561 in AK1 genomes (Supplementary Tables S1, S2). To determine if m6dA prefers certain chromosomes, we determined the ratio of m6dA marks versus unmethylated deoxyadenosines (dA) per chromosome (Fig. 1a). Overall, the m6dA/dA ratios obtained by SMRT-seq are as expected (0.00428% CHM1, 0.00464% AK1), as they lie within a similar range as previously reported for higher eukaryotes^45^. For the majority of chromosomes (chromosomes 1–14), the m6dA/dA ratio is almost identical between CHM1 and AK1 cells. We noticed a larger variation for some of the smaller chromosomes (16–22), which seems to correlate with smaller size. Interestingly, the m6dA/dA ratio is reduced in both human sex chromosomes in contrast to autosomes, in both cell lines (Fig. 1a). This is in contrast to what was reported for mice, where a higher presence of m6dA was detected on the X chromosome^16^. This might be explained by species differences. We next asked if there is a specific distribution of the m6dA along the chromosomes. In general, m6dA seems to be distributed approximately evenly over each chromosome (Supplementary Fig. S1).

We next determined the m6dA/dA ratio within different genomic locations and found a similar m6dA distribution within our genome features between the unrelated CHM1 and AK1 human genomes, generalizing our human findings (Fig. 1b). In both, we find that m6dA is significantly depleted in intergenic regions, but enriched in introns, exons, in the 5′ UTR and near transcriptional start sites (TSS) (Fig. 1b, Supplementary Fig. S2), confirming previous findings^13,16^. We also noticed that if a m6dA mark occurs within a gene feature, in most cases, it is not present in other gene features within the same gene (Supplementary Fig. S3). Notably, N-6 adenosine methylation in mRNA is known to be enriched in 3′ UTR regions, and not in 5′ UTR regions where m6dA is instead enriched^46^. This suggests that m6dA regulation and function differs from its RNA version. We next asked what the proportion of m6dA is within different gene regions. We noticed a high proportion of introns and intergenic regions have m6dA, in both cell lines (Fig. 1c, Supplementary Tables S3, S4). To analyze m6dA regions in more detail and context, we predicted which sequence context is enriched for m6dA. We find that for both CHM1 and AK1 genomes we see an enrichment for the AG/GA motif (Fig. 1d), consistent with previous studies^13,16,23^.

We also detected regions containing multiple m6dA, forming clusters. We define clusters as regions of 500 base pairs (bp) containing at least 10 m6dA. A significant number of m6dA, 11% and 5% of m6dA were found in such clusters for AK1 and CHM1 respectively (P-value < 0.001, Permutation test, Supplementary Fig. S4). Most of the m6dA within clusters are very close, with the median distance between m6dA marks of 4 base pairs for both cell lines. We found 230 such clusters for CHM1 and 514 for AK1. Together, these clusters contain 3,467 m6dA in CHM1 and 8,926 in AK1. We noticed that such regions have enriched A/T content of median 67% in CHM1 and 68% in AK1. In both cases, the sequence context surrounding the m6dA is repetitive. Although this could be a biological phenomenon, we cannot exclude that detecting m6dA within such highly repetitive regions may be a limitation of sequencing technologies and alignment limitations.

### m6dA heterogeneity between different human cell types

**Figure 2:**
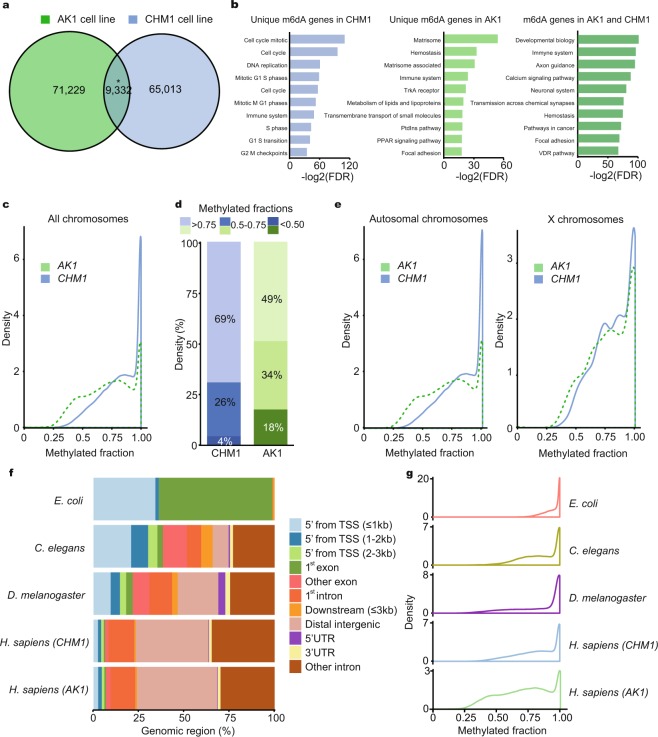
m6dA heterogeneity between different genomes and within cell populations. (**a**) Number of identified m6dA sites in () CHM1 and () AK1 cell lines. A significant overlap of precise m6dA nucleotide locations was identified (*P-value < 0.001, Permutation test). (**b**) Gene pathways that are enriched for m6dA. Pathways unique to m6dA genes in AK1 and CHM1 cell lines are shown, as well as pathways that are associated with genes that show m6dA in both AK1 and CHM1. (FDR) False discovery rate. (**c**) Density of m6dA methylated fraction. () CHM1, () AK1 cell lines. (**d**) Quantiles of m6dA methylated fraction. () CHM1, () AK1 cell lines. (P-value < 2.2 × 10^−16^, Wilcox test). (**e)** Density of m6dA methylated fraction, distinguishing autosomal and X chromosomes. () CHM1, () AK1 cell lines. (**f**) m6dA distribution within different genomic regions in different organisms. (TSS) transcriptional start site, (UTR) untranslated regions. (**g**) Density of m6dA methylated fraction in different organisms.

Identifying the level of heterogeneity of m6dA is required for elucidating m6dA function^41^. Hence, we asked how heterogenous m6dA is across our different human cell types. Our CHM1 and AK1 datasets identified 9,332 precisely overlapping m6dA nucleotide sites (P-value < 0.001, Permutation test, Fig. 2a, Supplementary Table S5). When adding 250 bp on either side of the m6dA mark, we identified an even larger overlap, 10,302 500 bp regions comprising of 16,856 in CHM1 and 15,089 m6dA in AK1. We next identified 10,232 genes with m6dA in CHM1 (Supplementary Table S6) and 11,565 genes with m6dA in AK1 genomes (Supplementary Table S7). Between the two samples we identified 7,677 genes that have m6dA in both CHM1 and AK1 (Supplementary Table S8). 58.8% of the exact matches in the samples occurred in gene regions, which is significantly greater than one would expect by chance (P-value < 2.2 × 10^−16^, Binomial test). Genes with m6dA in both cell lines were enriched for gene ontology (GO) terms such as pathways associated with neurons (Fig. 2b). Since both cell lines are not of neural origin, the function of m6dA on such genes in non-neural settings remains to be specifically investigated. When considering non-overlapping m6dA, cell specific pathways are enriched, such as hemostasis for the lymphoblastoid cell line AK1 found in blood, and cell cycle associated pathways for the CHM1 cell line (Fig. 2b, Supplementary Tables S9–S11). This function specific m6dA enrichment suggests involvement of m6dA in differentiation and maintenance of these cells.

### m6dA heterogeneity within the same cell population

Next, we asked how heterogenous m6dA is within a cell population. SMRT-seq DNA libraries were prepared from genomic DNA isolated from a population of cells. These libraries contain unamplified, original genomic DNA fragments. Hence, in SMRT-seq, when different DNA fragments are sequenced covering the same genome region, they have to originate from a homologous chromosome or a different cell. To determine m6dA heterogeneity, we identified SMRT-seq fragments that overlap the same genomic region and asked how many of them contained or did not contain m6dA at each precise position. This ‘methylated fraction’ reveals how often m6dA occurs at a precise position in the genome. For example, a 75% methylated fraction indicates that 75% of the same genomic region have m6dA at precisely the same location. This reveals how heterogenous m6dA is within a cell population.

We determined methylated fractions for our CHM1 and AK1 cell lines genome wide (Supplementary Tables S12, S13). In both cell lines we observe a high prevalence of a 100% methylated fraction (Fig. 2c,d). For the diploid cell line AK1 10% (8,065) of m6dA detected had 100% methylated fraction where for the haploid CHM1 cell line 20% (14,897) of all m6dA had 100% (Fig. 2c,d). This indicates that in these corresponding regions, m6dA is homogeneous as it occurs on all the corresponding DNA fragments. However, we also observe that m6dA is heterogeneous in many DNA regions when comparing CHM1 to AK1 (P-value < 2.2 × 10^−16^, Kolomogorov-Smirnov, Fig. 2c,d). The first quantile for AK1 was 48.8% whilst CHM1 had a first quantile value of 69.2% and had overall higher levels (P-value < 2.2 × 10^−16^, Wilcox test) (Fig. 2d). For both CHM1 and AK1 we find significant differences between the distribution of the methylated fraction in autosomal versus X chromosomes (Fig. 2e). CHM1 has more DNA fragments that are homogeneously methylated which could be due to CHM1 being a haploid cell line. Since we identified potential m6dA clusters as described above, we also asked what fraction is specifically methylated in these clusters. The m6dA methylated fraction is significantly lower within m6dA clusters (by 20% for CHM1 and 24% for AK1, P-value < 2.2 × 10^−16^, Wilcox test) as opposed to m6dA that do not occur in such clusters. This suggests that if this m6dA cluster is biologically relevant, some m6dA signal or ‘m6dA’ cloud might be required somewhere within a cluster region, but that the precise m6dA location within a region might not be important. Alternatively, it could suggest that the m6dA cluster signal, which is embedded in highly repetitive sequences, might not be detected reliably by SMRT-seq due to its highly repetitive nature.

To correlate m6dA methylated fractions with potentially different roles, we determined the methylated fractions and m6dA genomic features for different organisms (Fig. 2f,g). In these organisms, differences in m6dA abundance, motifs and distribution was previously reported^10–22^. We compared our SMRT-seq CHM1 and AK1 human data to published SMRT-seq datasets from bacteria^47^, worms^12^ and flies^47^. Since no fly SMRT-seq dataset has so far been analyzed for the presence of m6dA, our analysis also provides the first genome wide analysis of m6dA at nucleotide resolution within the *Drosophila* genome. We identified m6dA sites in *E. coli* (37,279), *C. elegans* (17,555) and *D. melanogaster* (14,619) and determined their genomic features and methylation fractions (Fig. 2f,g, Supplementary Table S14). The bacterial genomes are very small, lack introns and have short non-coding regions, explaining why most m6dA sites fall within exons and regions upstream of TSS sites. In worms m6dA is found in intronic and TSS upstream regions. In flies, we find m6dA less prevalent in promoters and increasingly enriched in intergenic regions. In humans CHM1 and AK1 cell lines, m6dA enrichment in intergenic regions is the most abundant group while it is much less prevalent in promoters and coding regions (Fig. 2f). Overall, our analysis implies that with increasing organism complexity, m6dA shifted towards intergenic regions, away from promoters and coding regions. Comparing these distributions with the methylated fractions in different organisms, we encounter the highest m6dA homogeneity in bacteria (Fig. 2g). This could be due to the fact that bacteria are haploid, have fewer non-coding regions and their m6dA motif is precisely defined due to its role in their immune response^4–9,22,47^. In contrast, worms and flies show a wider methylation fraction distribution, while humans show the broadest distribution while maintaining a high 100% methylated fraction (Fig. 2g). Overall, our analysis across species shows that m6dA differs between species both in their genomic distribution and in their methylation fraction. This further corroborates that different m6dA methylated fractions or level of heterogeneity are likely to be associated with different genomic features, molecular mechanisms and biological functions. Since our human datasets show a high prevalence of a 100% methylated fraction, but also a wide distribution, it implies that human m6dA with a high homogeneity could have a distinct role.

### m6dA heterogeneity between chromosome pairs

Epigenetic marks can differ between individual humans, cell types, but also between two chromosome pairs within one cell^48,49^. The latter can result in X chromosome inactivation^50,51^ and genomic imprinting^52^. Genomic imprinting describes a process in which homologous genes are expressed in different ways, depending on whether they are inherited from the mother or the father. In the case of X inactivation, one of the two X chromosomes in female mammals is transcriptionally silenced, which causes significant genome wide transcriptional changes^51^. Understanding the regulation of epigenetic differences on the two chromosome pairs is important: if epigenetic marks are not correctly placed this can result in disease^48,49,52^. The DNA modification m5dC plays a key role in regulating differential expression from homologous chromosomes, parental imprinting and X-chromosome inactivation^48,52^. Hence, we investigated if m6dA could also be differentially methylated between chromosome pairs in the diploid AK1 cell line.

**Figure 3:**
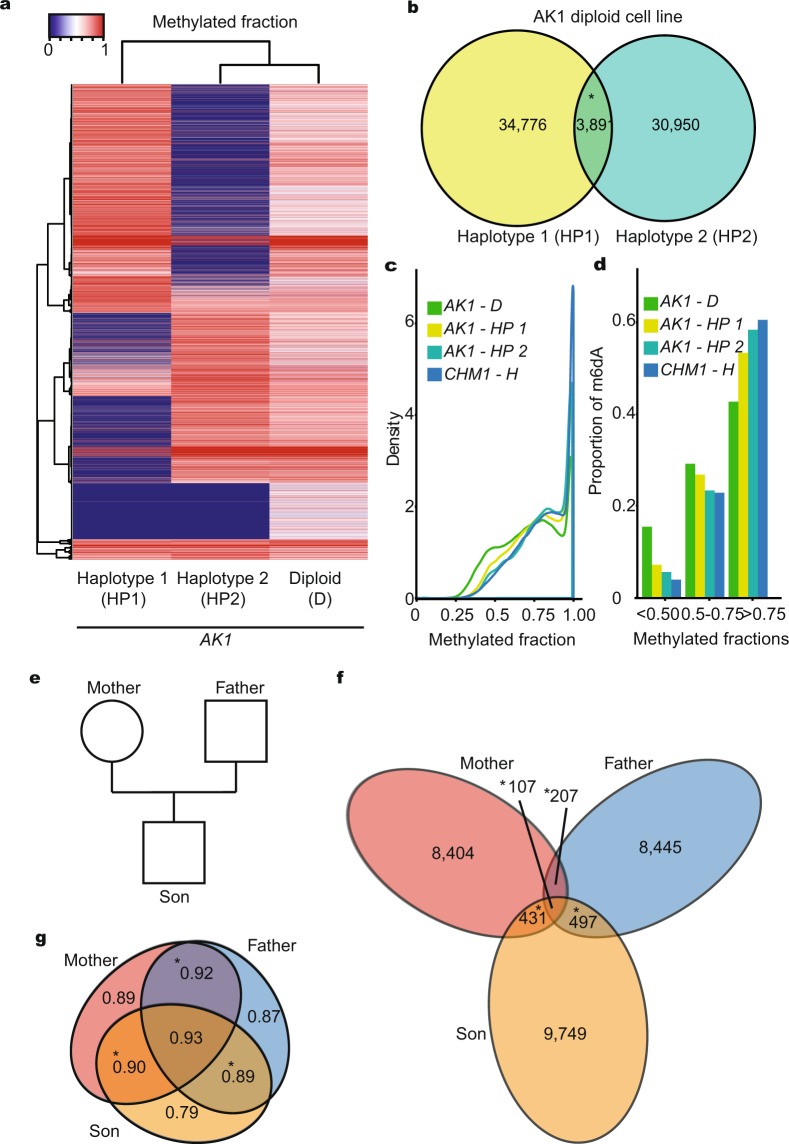
m6dA heterogeneity within one chromosome and chromosome pairs. (**a**) Heatmap of m6dA methylated fraction for identified m6dA sites in the diploid and haploid AK1 genomes. (HP1) Haplotype 1, (HP2) Haplotype 2, (D) Diploid. () Low methylated fractions, () high methylated fraction. (**b**) Overlap between the number of m6dA identified on each haplotype of AK1 (HP1) Haplotype 1, (HP2) Haplotype 2 (*P-value < 0.001, Permutation test). (**c**) Density of m6dA methylated fraction for the diploid (), haploid ( and ) AK1 and haploid CHM1 () genomes. (HP1) Haplotype 1, (HP2) Haplotype 2, (D) Diploid, (H) Haploid. (**d**) Proportion of low, mid and high m6dA methylated fractions for the diploid (), haploid ( and ) AK1 and haploid CHM1 () genomes. (HP1) Haplotype 1, (HP2) Haplotype 2, (D) Diploid, (H) Haploid. (**e**) Illustration of family tree in which m6dA was identified within the genome from lymphoblastoid cells. (**f**) Number of overlapping m6dA within family. (*P-value < 0.001, Permutation test). () Mother, () father, () son. (**g**) Median methylated fraction overlapping within family (*P-value < 0.05, Wilcox test). () Mother, () father, () son.

First, using single nucleotide polymorphisms (SNPs), we separated the SMRT-seq AK1 sequencing reads into haplotypes, which are chromosome regions that are located on the same chromosome (Supplementary Fig. S5). We were able to separate 72% of our genome into distinct haplotypes (Supplementary Table S15). Second, for each haplotype we identified 38,667 and 34,841 m6dA marks, at coverage >20 and IPD ratio ≥4 (Fig. 3a, Supplementary Figs S6, S16). Strikingly, we found that the majority of m6dA were present only in one haplotype (Fig. 3a). To investigate if the m6dA methylated fraction differs between chromosome pairs, we compared the methylated fraction of the diploid AK1 cell line to its haploid components and also to the CHM1 cell line that is biologically haploid (Fig. 3c,d). We noticed that the distribution of the methylated fraction for the individual haplotypes is closer to the haploid CHM1 cell line than to the diploid AK1 dataset (Fig. 3c,d). This could imply that ploidy could have an impact on the heterogeneity of the m6dA, although cell type differences could also explain this result. We also found that the methylation fraction was higher on individual haplotypes than on the combined diploid dataset (Fig. 3d). Since different haplotypes originate from different chromosomes, different methylation fractions can be observed between chromosome pairs. This demonstrates that some deoxyadenosines (dA) are consistently differentially methylated between chromosome pairs. This could suggest that m6dA is genotype dependent and is regulated in cis, and that m6dA could have a role in imprinting, X chromosome inactivation or be inherited from parental chromosomes.

To gain insight into m6dA heritability within a family, we used publicly available SMRT-seq dataset from the genome of one family with one mother, father and their son (Fig. 3e)^53^. The genome of all family members was isolated from the same cell type, lymphoblastoids, in which we identified specific m6dA sites. We found that there is significant overlap between all pairs of family members (P-value < 0.001, Permutation test). We identified 314 m6dA sites that overlap at nucleotide resolution between mother (3.4%) and father (3.3%) (Fig. 3f,g, Supplementary Table S17). The low number of overlaps is due to lower data coverage of all family members. Nevertheless, the overlap in the parents, who are genetically not related, is significantly more than one would expect at random (P-value < 0.001, Permutation test), which suggests m6dA conservation in humans. 538 m6dA locations precisely overlapped between the son and the mother (P-value < 0.001, Permutation test), and 604 m6dA between the son and the father (P-value < 0.001, Permutation test) (Fig. 3 f,g). We find that the overlap between father or mother and son is significantly more conserved (P-value 7.4 × 10^−8^ for mother and son, P-value 1.2 × 10^−13^ for father and son, Proportion test) than the overlap between father and mother, who are genetically not related. This suggests m6dA conservation and inheritance within families.

### m6dA and transcription

**Figure 4:**
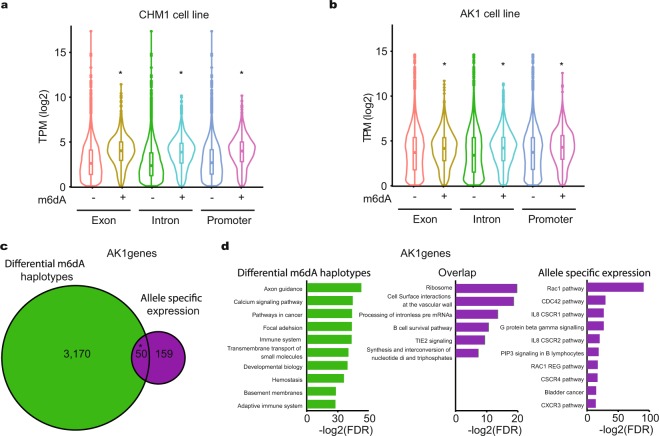
m6dA and gene transcription. (**a**) CHM1 cell line gene expression from gene regions with (+) and without (−) m6dA identified with SMRT-seq. Transcripts are significantly higher in exons, introns and promoters with m6dA (*P-value < 2.2 × 10^−16^, Wilcox test). (TPM) transcripts per million. (**b**) AK1 cell line gene expression from gene regions with (+) and without (−) m6dA identified with SMRT-seq. Transcripts are significantly higher in exons, introns and promoters with m6dA (*P-value < 2.2 × 10^−16^, Wilcox test). (TPM) transcripts per million. (**c**) Overlap between genes with differentially methylated haplotypes and genes with allele specific gene expression in AK1 cells (P-value 9.1 × 10^−15^, hypergeometric test). (**d**) Pathways enriched for genes with differentially methylated haplotypes, genes with allele specific gene expression and their overlap in AK1 cells. (FDR) False discovery rate.

To investigate if there is a link between m6dA and gene expression, we carried out RNA high throughput sequencing (RNA-seq) on CHM1 cell lines and used published AK1 RNA-seq data^43^ (Supplementary Fig. S7, Supplementary Tables S18, S19). This allowed us to correlate our m6dA containing genes with gene expression. We distinguished between gene and gene features that are and are not associated with m6dA and correlated these with our RNA-seq datasets. We found that gene regions with m6dA are associated with higher RNA levels (transcripts per million, TPM) than genes which do not have m6dA (Fig. 4a,b). Specifically, we noticed that the number of low expression transcripts is reduced when m6dA is present, irrespective of the cell type (Fig. 4a,b, Supplementary Tables S20, S21). We also carried out DNA immunoprecipitation followed by sequencing (DIP-seq) with an antibody against m6dA in matching biological samples^13,14^, which corroborated our SMRT-seq findings (Supplementary Fig. S8, Supplementary Tables S22, S23). Overall, it appears that m6dA promotes transcriptional expression as reported by others^16,23^, in contrast to one report in mice^16,23^, which remains to be confirmed.

Given the variation in m6dA across haplotypes we sought to identify a corresponding relationship between m6dA and gene expression for different haplotypes. To do this, we generated allele specific RNA-seq counts for each gene to identify genes with allele-specific expression (Supplementary Table S24)^54^. We found 50 genes with allele specific expression showing differential methylation, which are linked to ribosomal, processing of intron-less pre-mRNAs and B cell survival pathways (P-value 9.1 × 10^−15^, Hypergeometric test, Fig. 4c,d, Supplementary Tables S25–S28). We identified genes such as KDM4B, TBP, TCF4 and PEG10. KDM4B demethylates histones H3K9me^2^ and H3K9me^3^ that are enriched in constitutive heterochromatin, and as such permits access to various transcription factors^55^. TBP is a TATA box binding protein that is required for RNA polymerase II function^56^. Since KDM4B and TBP are differentially methylated and show allele-specific expression, this suggest that m6dA could be involved in their regulation, and as such might have also wide-ranging and indirect, downstream effects on other genes. We also find TCF4, which is an E-box binding transcription factor, that is particularly important in the brain where its haploinsufficiency has been associated with mental retardation^57^. Hence, investigating whether m6dA plays a role in TCF4 mediated haploinsufficiency is of great importance. Bearing in mind the association of m6dA with neuronal pathways (Fig. 2b), m6dA might influence an array of neuronal genes, which is likely to occur in a chromosome specific manner. As such, the presence of m6dA on one chromosome alone could fine-tune gene expression during brain development or memory formation. This fine-tuning could lead to different cell identities and as such contribute to the vast diversity of expression profiles, brain cell types and brain network. Also, since it appears that m6dA could be imprinted or inherited within families, m6dA misregulation in neuronal genes could be also the cause of some brain or mental disorders that are inherited within families. Also, we identified PEG10, which is a known paternally-expressed imprinted gene that is derived from retrotransposons, which suggests that m6dA could also be involved in imprinting^58^. Overall, m6dA could be involved in regulating fundamental biological pathways, highlighting the importance of studying m6dA and its potential influence on human disease.

## Discussion

In DNA modifications play important functions in the human genome, for example by regulating transcription. As such, it is not surprising that their misregulation can result in human diseases^1–3,59^. In particular, m5dC heterogeneity is prominent in diseases such as human cancers. Hence, to improve treatments, a better understanding of epigenetic heterogeneity is important^41^.

m6dA has only recently been discovered in vertebrates and mammals^13^. Despite its low abundance in eukaryotes, m6dA appears to be biologically relevant. Further, since changed m6dA levels are associated with human diseases, it is essential to study m6dA in humans. For example, higher m6dA levels were reported in brain cancer glioblastomas and decreasing m6dA lead to lower tumour cell proliferation^24^. This not only suggests that misregulation of m6dA levels may result in disease, but that changing levels of m6dA could alleviate or in future even cure certain diseases. Hence, studying m6dA opens up novel future treatment opportunities for human cancers.

Since our study also suggests m6dA conservation and inheritance within families, taken together it implies that alterations of m6dA could predispose to human diseases like cancer. But it is not only cancer m6dA is associated with. m6dA was also found at decreased levels in type two diabetes mellitus^15,21,24^. Interestingly, we also identified an association between m6dA and neuronal genes, many of which are key genes involved in human neurological disorders. A recent independent study found that upon environmental stress, there is an increase in m6dA levels in mouse brain. Specifically, any m6dA stress-induced changes significantly overlapped with genes that are associated with neuropsychiatric disorders^17^. In addition, our study also identified 50 genes with allele specific expression showing differential m6dA, such as TCF4. Since TCF4 is important in the brain where its haploinsufficiency has been associated with mental retardation^57^. As such, studying m6dA opens up a myriad of future opportunities to not only better understand a myriad of diseases, but to explore m6dA as a future therapeutic tool.

To be able to study human diseases and develop tools, we need to precisely understand m6dA characteristics. Here, for the first time, we demonstrate that despite low m6dA abundance, the majority of the m6dA mark occurs consistently at a precise nucleotide location. This high degree of m6dA homogeneity within a cell population and within a chromosome, suggests that m6dA is tightly regulated. This is important, as it demonstrates for the first time that m6dA is precise, hence, is tightly controlled and as such could represent a unique drug target for future human disease therapies.

Additionally we identified sites with heterogenous m6dA, where the methylation fraction seems to be lower. This suggests that m6dA could have different functions in a cell population or in cells, depending on their heterogeneity and location. Although m6dA seems to have a role in transcriptional regulation, which could impact human diseases, to us it appears that m6dA might have also other roles that are differently regulated.

Overall, our analyses demonstrate for the first time the precision and consistency by which the m6dA mark occurs within the human genome, suggesting that m6dA marks are precisely inherited in humans, are chromosome specific and could have a role in imprinting. In future, this high degree of m6dA precision and homogeneity could open up alternative routes to target specific m6dA regions for drug treatment therapies.

## Methods

### Cell lines

The CHM1 cell line was obtained from Prof Evan Eichler, using their published protocol^44^. Cell lines have been genotyped and tested for mycoplasma contamination by qPCR and the LookOut Mycoplasma Detection kit (Sigma).

### Genomic DNA isolation

DNA was isolated following our published protocol^14^.

### RNA isolation

Cells were processed with the RNeasy Kit (Qiagen). DNA was eliminated following the manufacturers guidelines (RNase-Free DNase for use with RNeasy Columns, Qiagen).

### DNA & RNA quality control

Size distribution and quality of (intact or fragmented) DNA and RNA was monitored using TapeStation, following manufacturer’s protocols (Agilent).

### DNA immunoprecipitation (DIP)

DNA immunoprecipitation was prepared following our published protocol^14^. A m6dA recognizing antibody from Synaptic Systems GmbH was used (Cat. No. 202011). Each experiment consisted of one DIP and its corresponding input control.

### Illumina sequencing library preparation

DNA libraries were prepared using the TruSeq Nano DNA Library Prep Kit and following the manufacturer’s protocol (Illumina). RNA libraries were made with the TruSeq Stranded Total RNA Kit using the manufacturers protocol (Illumina). All our Illumina DNA libraries were sequenced using an Illumina HiSeq 4000 instrument in single end, 50 bp mode. Our RNA libraries were sequenced in paired end, 150 bp mode. Three biological replicates (separately grown tissue culture cells) were processed. RNA and DNA libraries were matched, originating from the same biological material.

### SMRT data processing

SMRT reads were obtained from the SRA database (Supplementary Table 29 for accessions). Adaptors were trimmed from h5 files using bax2bam (version 0.0.8). Reads were aligned using blasr (version 5.3) to the UCSC hg38 (GRCh38/hg38) human genome with parameters bestn 1, minRawSubreadScore 750, minReadLength 500, and minSubreadLength 50. m6dA were identified and methylated fractions calculated using ipdSummary (version 2.3, mapQVthreshold 50). m6dA were filtered to have at least 20x coverage and an IPD ratio > 4.

### Family SMRT data processing

Data from the GenomeInABottle consortium was obtained from https://github.com/genome-in-a-bottle/giab_latest_release using the Ashkenazim Trio. Only reads sequenced using the P6-C4 chemistry were used for each sample. This gave a coverage of 27x for the mother, 29x for the father and 67x for the Son. As SMRT-seq is strand specific the coverage filters we used for these data sets were 14x for the mother, 15x for the father and 33x for the son. All m6dA were filtered to have IPD ratio > 4. The significance of the overlaps of m6dA location between family members was determined using a permutation test. To determine if there was a greater overlap between related individuals the differences in m6dA overlaps were assessed using the proportions test. For the Permutation test, random sets of m6dA for each family member were selected from those that did not pass initial coverage or IPD ratio. The overlap between 1,000 random sets was calculated and compared to the amount of overlap observed between m6dA passing filters. To determine if there was a greater overlap between related individuals the differences in m6dA overlaps were assessed using the proportions test (Pearson’s Chi-squared test) in R stats package (version 3.4.2). To compare the overlap between mother and father and the mother and son, the proportion of m6dA identified for the mother overlapping between mother and father was compared to the proportion of m6dA in the son overlapping between the mother and son. This allows the test to account for the relatively larger number of m6dA detected overall for the son. The analogous test was also performed between the father and mother and the father and son.

### m6dA annotation

The UCSC centromere track was obtained through rtracklayer (version 1.36.6) and was used to annotate m6dA. Each chromosome was split into 200 equal bins. The proportion of m6dA/dA was calculated for each bin. Annotations were provided by bioconductor packages Org.Hs.eg.db (version 3.4.1) and transcript annotations in known genes from TxDb.Hsapiens.UCSC.hg38.knownGene (version annotation 3.4.0). For m6dA/dA the number of dA was determined from sequences in Bsgenome.Hsapiens.UCSC.hg38 (version 1.4.1). All m6dA were annotated using the ChIPseeker R package (version 1.6.7). Promoter regions were defined as 1 kb upstream from the transcription start site. Enrichment scores for m6dA within each genomic region were calculated by permutation test.

### Motif detection

ipdSummary provides the sequence context 20 bp upstream and downstream of a methylated adenine. These sequence contexts were used to search for motifs. The sequences up and downstream of all methylated adenines were matched to all possible dinucleotide sequences. The significance of sequences was determined by the Fisher’s exact test. 4-mers with overlapping dinucleotide sequences were combined to give a combination of overlapping sequences and the significance was tested using the Fisher’s exact test. The null set for the Fisher’s exact contingency table was determined by using m6dA identified from the PacBio sequencing that did not pass the 20x coverage and IPDratio filters. This allowed for the elimination of part of the biased detections at motifs. The null set m6dA had at least 11x coverage which allowed for detection of m6dA and calculation of the methylated fraction.

### Cluster analysis

For the m6dA cluster analysis we refer to m6dA in clusters as cluster m6dA and those not within a cluster as single m6dA. Cluster significance was determined by a Permutation test used to calculate empirical p-value for the significance of the m6dA found in 500 bp regions. To test for differences in the distribution of cluster and single m6dA a proportion test was used. First, the Proportion that was defined as cluster m6dA within each genomic region was calculated. Second, the proportion of single m6dA across different genomic regions was calculated. The proportion test was then used to test for differences in the deposition of m6dA in genomic regions depending on whether they are defined as cluster m6dA or single m6dA.

### Differential methylation

To test for differential methylation of genes between samples the total number of methylated cells was defined by multiplying the methylated fraction by the coverage at the m6dA position. For each gene this was summed across all dA positions methylated in either sample. The difference in the proportion of methylation between the two samples was assessed using the proportions test in the R stats package (Pearson’s Chi-squared test).

### Haplotyping reads

We used the Illumina data to call variants for the AK1 data sets using the GATK pipeline (v.3.4) and filtered using SnpEff (version 4.3i) for quality scores and coverage. The SMRT-seq reads were used to phase variants using WhatsHap (version 0.17), producing a phased vcf file. These phased variants were then used to tag the SMRT-seq reads according to their haplotype also using WhatsHap. m6dA for the haplotyped reads were identified using ipdSummary and the same settings as for the full data set. To determine the coverage at non-methylated adenines in a sample bedtools genomecov (-strand, -bg) was used (version 2.25.0). For hierarchical clustering heatmap3 (version 1.1.1) was used to cluster by sample (H1, H2, diploid) and by m6dA (rows), using euclidean distance between methylated fractions. m6dA were included that were found in the diploid analysis and where 20x coverage was obtained for each haplotig. A linear regression model (lm function R stats, version 3.4.2) was used to regress the diploid methylated fraction to the methylated fraction for both haplotigs. Significance of regression parameters was determined by t-test. For the diploid data after calling variants this data was read into R using readVcf in the VariantAnnotation (version 1.22.3) package and genomicRanges (version 1.28.6) was used to determine distance to the closest SNP.

### Statistical tests

For significance tests, the location of the m6dA were randomly shuffled using the shuffle function in the R package ChIPseeker. m6dA were shuffled 1,000 times giving a minimum p-value of 0.001. For clusters, the number within 500 bp of another m6dA mark was calculated to give an empirical null distribution. Overlaps between m6dA data sets were done using findOverlaps and countOverlaps from the GenomicRanges package in R and the significance was determined using the Permutation test. For enrichment analysis, two gene sets were obtained from GSEA, the hallmark set (h.all.v6.2.symbols.gmt) and canonical pathways set (c2.cp.v6.2.symbols.gmt). These were read into R using the qusage package (version 2.10.0). Significance of the overlap between genes and the GSEA databases were tested using Chi-square test.

### RNA-seq data analysis

All RNA-seq data was paired end and was mapped following ENCODE guidelines using STAR (version 2.5.0a) to the UCSC GRCh38/hg38 genome. Mapped reads were filtered to have minimum mapping quality 20 and were summarised into gene level counts using Rsubread (version 1.26.1). A GTF file for annotation was obtained from Gencode (GRCh38, version 28, Ensembl 92). For transcripts per million calculations the annotation and transcript lengths were taken from Rsubread. Our own sequencing was carried out with 3 biological replicates, indicated by n.

The phased vcf file for AK1 (generated from the Illumina and SMRT-seq data as outlined above) was used to phase Illumina RNA-seq reads using PhASER (version 0.9.9.4) with the haplotig and region blacklists (for hg38 obtained from https://github.com/secastel/phaser/tree/master/phaser). PhASER was also used to generate gene level counts for each phased allele. RPKMs for the full data set (not phased) were generated using the rpkm function in edgeR (version 3.18.1) and gene lengths from Rsubread. For genes with allele specific counts, significantly different allele expression was determined using a binomial test in R. P-values were corrected using Benjamini-Hochberg.

### DIP-seq data analysis

For quality control, we had statistics for the non-redundant fraction, cross-correlation values and the SPP statistics generated from run_SPP.R. Three replicates were processed using macs2 to call peaks at a relaxed p-value threshold of 1 × 10^−3^. These peaks were then filtered according to multiple hypothesis corrected p-values. Overlaps between replicates were calculated using find Overlaps in the GenomicRanges package. Overlapping peaks between replicates were merged and the overlap between the SMRT-seq data set was calculated and significance assessed through random perturbation using the regioneR package (version 3.8).

### Species analysis

SMRT-seq datasets were processed as outlined above. Annotation and plotting of genome regions was done using ChIPseeker (version 1.6.7), with promoters defined as 1 kb upstream of the TSS. For the different species the following annotation packages were used; *D. melanogaster* (org.Dm.eg.db and TxDb.Dmelanogaster.UCSC.dm6.ensGene, version 3.4.1), *C. elegans* org.Ce.eg.db and TxDb.Celegans.UCSC.ce6.ensGene (version 3.2.2), *E. coli* org.EcK12.eg.db and TxDb was generated from *E. coli* GFF (*NCBIAssembly*: *GCF*_000005845.2) using makeTxDbFromGFF in GenomicFeatures (version 1.28.5).

### Accession Codes

Newly generated data, such as RNA-seq data and m6dA DIP-seq data, has been deposited in the Gene Expression Omnibus database under Accession Code GSE123065. We also used published datasets that are listed in the Supplementary Table S29.

## Supplementary information

Supplementary Figure S1-S8

Supplementary Table S1-S29
