# Supplementary material for: Characteristics and homogeneity of N6-methylation in human genomes
Source: Sci Rep. 2019 Mar 26;9:5185. doi: 10.1038/s41598-019-41601-7 (PMC6435722; doi:10.1038/s41598-019-41601-7)
Supplement: Supplementary file 1 — Supplementary Figure S1-S8 [file 41598_2019_41601_MOESM1_ESM.docx]

**Characteristics and homogeneity of N6-methylation in human genomes**

**Clare E. Pacini**1,2**, Charles R. Bradshaw**1**, Nigel J. Garrett**1,2**, and Magdalena J. Koziol**1,2*

1Wellcome Trust Cancer Research UK Gurdon Institute, University of Cambridge, Cambridge, CB2 1QN, UK

2Department of Zoology, University of Cambridge, Cambridge, CB3 3EJ, UK

*Correspondence and requests for materials should be addressed to M.J.K. (email: [mjk39@cam.ac.uk](mailto:mjk39@cam.ac.uk))

**Supplementary Figure S1**

**Chromosome location of m6dA.** (**a**) m6dA chromosome location of CHM1 cell line. () Centromeric regions, () m6dA in centromere, () m6dA not in centromere, () size increases with m6dA/dA ratio. (**b**) m6dA chromosome location of AK1 cell line. () Centromeric regions, () m6dA in centromere, () m6dA not in centromere, () size increases with m6dA/dA ratio.

**Supplementary Figure S2.**

**Genome-wide distribution of m6dA in the vicinity of genes.** (**a**) Distribution of m6dA/dA in CHM1 cell line around TSS, from 2kb 5′ to 2kb 3′. (K) thousand, (TSS) transcriptional start sites. (**b**) Distribution of m6dA/dA in AK1 cell line around TSS, from 2kb 5′ to 2kb 3′. (K) thousand, (TSS) transcriptional start sites.

**
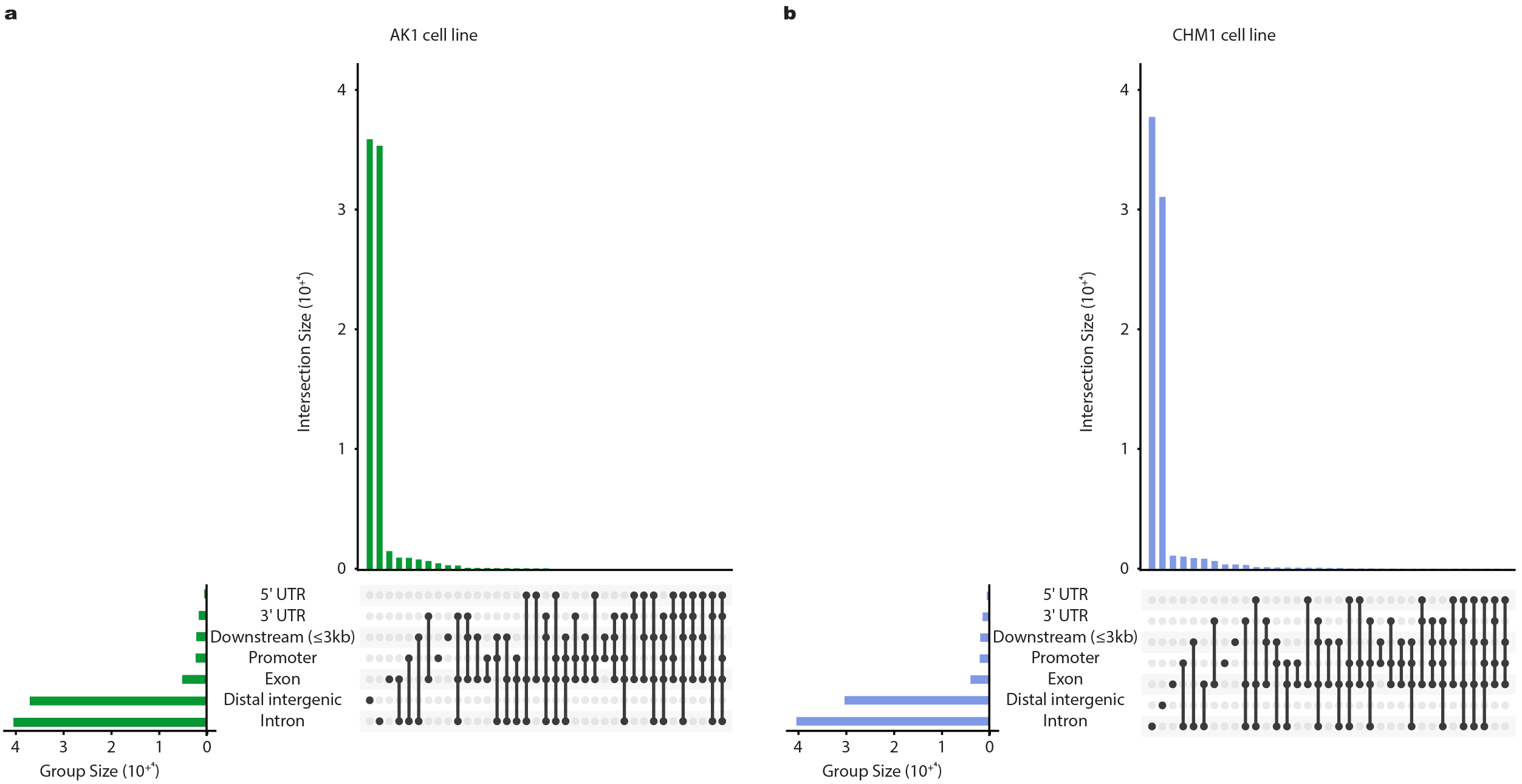
**

**Supplementary Figure S3.**

**Genomic location of m6dA. (a)** Abundance of m6dA in different genomic regions in AK1 cell line. Intersection size indicates where m6dA can be annotated in genome area denoted as () below graph. Multiple per column indicate presence of m6dA in more than one region of the same gene. Group size indicates total abundance of m6dA annotation in genomic region, irrespective if m6dA also occurs in another region within the same gene. **(a)** Abundance of m6dA in different genomic regions in CHM1 cell line. Intersection size indicates where m6dA can be annotated in genome area denoted as () below graph. Multiple per column indicate presence of m6dA in more than one region of the same gene. Group size indicates total abundance of m6dA annotation in genomic region, irrespective if m6dA also occurs in another region within the same gene.

**
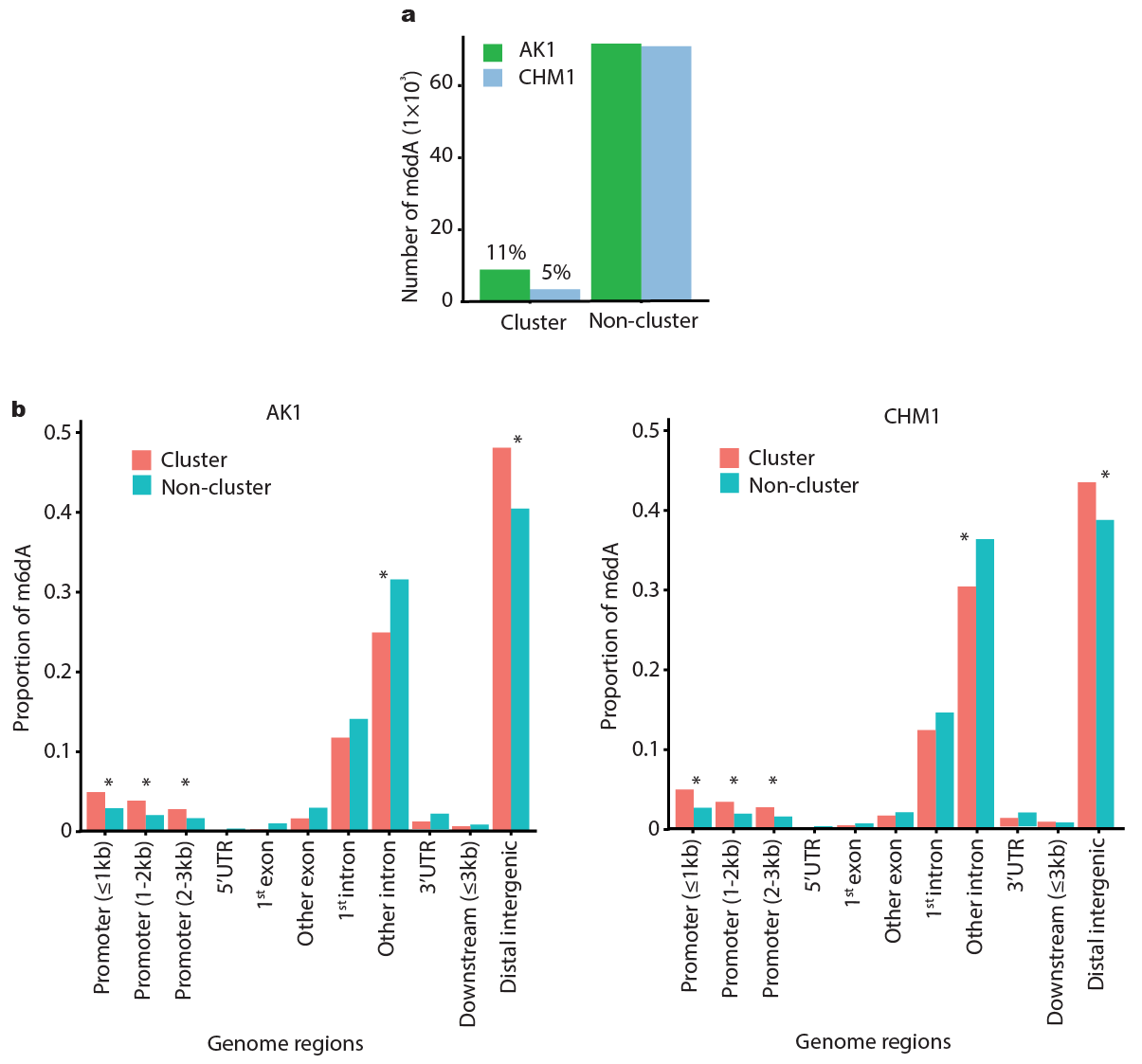
**

**Supplementary Figure S4­.**

**Genomic location of m6dA. (a)** () AK1 and () CHM1 cell lines contain a significant number of m6dA within clusters (P-value <0.0001, permutation test). Clusters are defined as 500bp regions containing at least 10 m6dA. (**b**) Proportion of m6dA in distinct genomic regions for AK1 and CHM1 cell lines. () m6dA that occur only within m6dA clusters, ()m6dA not occurring in clusters (P* <2.4 x10^-9^, by binomial test on m6dA peaks). (UTR) untranslated regions.

**Supplementary Figure S5**

**Outline of the methodology for calling m6dA in different haplotigs.** (**a**) Illumina data is used to identify SNPs due to its higher accuracy. (**b**) SNP pairs are used to phase SNPs into sets of consistent haplotigs. The longer length SMRT-seq data is used here as more SNPs can be covered by a single read. (**c**) SMRT-seq aligned reads are tagged according to which haplotig they are from. (**d**) SMRT-seq aligned reads are split according to haplotig, generating two BAM files. (**e**) The separate BAM files are each run through the SMRT-seq m6dA detection pipeline, generating separate m6dA base calls for each haplotig.

**Supplementary Figure S6.**

**Circos plot showing m6dA haplotype specific distribution across the AK1 cell line genome.** From outside to inside: (i) Chromosome location. (ii) Smallest () and largest () haplotype size. Gaps within chromosome relate to regions that are difficult to map, such as centromeres or structural variants. (iii) () m6dA identified in diploid AK1 data set. (iv) () m6dA identified in one haplotype AK1 data set. (iv) () m6dA identified in one haplotype AK1 data set. Only 72% of genome represented, as only 72% of the genome could be separated into distinct haplotypes (Supplementary Table S15).

**Supplementary Figure S7.**

**Correlation of RNA-seq data from CHM1 cells.** Heat map of Pearson correlation coefficient values from comparisons between CHM1 cell line RNA-seq 3 biological replicates. Correlation was calculated pairwise for all samples, excluding windows in which both samples in the pair had zero depth. The colour in each plot reflects the correlation value (Pearson correlation coefficient), which is also shown for each comparison.

**Supplementary Figure S8.**

**M6dA and gene transcription.** (**b**) AK1 cell line gene expression from gene regions with (+) and without (-) m6dA identified with m6dA DIP-seq. Transcripts are significantly higher in introns and promoters with m6dA (*P-value <2.2x10^-16^, Wilcox test). The number of m6dA identified by DIP-seq in exons was small and may explain the lack of significant differences in this group. (TMP) transcripts per million.
